# Hops (*Humulus lupulus* L.) Bitter Acids: Modulation of Rumen Fermentation and Potential As an Alternative Growth Promoter

**DOI:** 10.3389/fvets.2017.00131

**Published:** 2017-08-21

**Authors:** Michael D. Flythe, Isabelle A. Kagan, Yuxi Wang, Nelmy Narvaez

**Affiliations:** ^1^USDA, Agricultural Research Service, Forage-Animal Production Research Unit, Lexington, KY, United States; ^2^Department of Animal and Food Sciences, University of Kentucky, Lexington, KY, United States; ^3^Department of Plant and Soil Sciences, University of Kentucky, Lexington, KY, United States; ^4^Agriculture and Agri-Food Canada, Lethbridge Research Centre, Lethbridge, AB, Canada; ^5^SGS Canada Inc., Agricultural Services, Guelph, ON, Canada

**Keywords:** antimicrobial growth promoter, phytochemicals, plant secondary metabolites, rumen microbiology, feed efficiency, alternatives to antibiotics

## Abstract

Antibiotics can improve ruminant growth and efficiency by altering rumen fermentation *via* selective inhibition of microorganisms. However, antibiotic use is increasingly restricted due to concerns about the spread of antibiotic-resistance. Plant-based antimicrobials are alternatives to antibiotics in animal production. The hops plant (*Humulus lupulus* L.) produces a range of bioactive secondary metabolites, including antimicrobial prenylated phloroglucinols, which are commonly called alpha- and beta-acids. These latter compounds can be considered phyto-ionophores, phytochemicals with a similar antimicrobial mechanism of action to ionophore antibiotics (e.g., monensin, lasalocid). Like ionophores, the hop beta-acids inhibit rumen bacteria possessing a classical Gram-positive cell envelope. This selective inhibition causes several effects on rumen fermentation that are beneficial to finishing cattle, such as decreased proteolysis, ammonia production, acetate: propionate ratio, and methane production. This article reviews the effects of hops and hop secondary metabolites on rumen fermentation, including the physiological mechanisms on specific rumen microorganisms, and consequences for the ruminant host and ruminant production. Further, we propose that hop beta-acids are useful model natural products for ruminants because of (1) the ionophore-like mechanism of action and spectrum of activity and (2) the literature available on the plant due to its use in brewing.

The purpose of this review is to collect and reexamine experiments that evaluated bitter acids from the hops plant (*Humulus lupulus* L.) as modifiers of rumen microbiology. These experiments were largely performed and reported over the last decade. However, historical work is drawn upon for context and for the origins of hypotheses. The thesis of the review is that the effects of bitter acids on rumen bacteria are similar to the effects of ionophore antibiotics, which have been used in ruminant nutrition for many years. This similarity and the vast body of current and historical literature on the hops plant make it an ideal model among rumen-active plant secondary metabolites. We have encountered a number of natural products researchers interested in microbiological uses of the bitter acids, but unfamiliar with rumen microbiology and its role in ruminant nutrition. Likewise, there are many ruminant scientists who are unfamiliar with the plant and its biochemistry. Both of these groups are the intended audience. Therefore, the review includes introductions to rumen microbiology and the hops plant.

## Introduction to Rumen Function

The rumen is the distinguishing adaptation of the ruminant animal. This first chamber of the digestive tract is, in some respects, more like an intestine than a gastric stomach (1). The ideal pH of the rumen is neutral, and it does not make the copious mucus produced by a gastric stomach to protect it from pepsin and acidic conditions. Like an intestine, the rumen epithelium absorbs certain nutrients, and it also serves as habit for a dense community of phylogenetically diverse microorganisms (2, 3). Unlike the microorganisms in the lower digestive tract, those found in the rumen gain metabolic access to the feed before the animal host. The trait of rumen microorganisms having first access to the feed has adaptive value because they in turn give the host metabolic access to fiber (1). Ruminants, like other mammals, do not make enzymes to catabolize cellulose and hemicellulose. However, the fibrolytic bacteria and fungi break down these fibers to the constituent sugars. The sugars are fermented and the fermentation acids can be absorbed through the rumen epithelium.

Fiber digestion is only one lifestyle of rumen microorganisms. Each feed component is a possible growth substrate, and thus, an ecological niche. A group of related niches are collectively called a *guild*, and it is in terms guilds or functional groups that we usually consider rumen microorganisms. In addition to the fibrolytic or cellulolytic bacteria, there are also those that utilize starch, pectin, or simple sugars to produce fermentation acids (3). Amylolytic bacteria can cause rumen acidosis when the dietary concentration of starch or water-soluble carbohydrates is too great (2). Many predominant amylolytic bacteria, such as *Streptococcus bovis*, exhibit homolactic fermentation. An excess of starch results in accumulation of lactic acid; the rumen pH declines; fiber digestion slows, and the animals develop problems ranging from feed refusal to rumen ulceration and death. Sub-acute rumen acidosis is a major problem in modern dairy operations.

Under normal conditions lactate production plays an important role in the rumen ecosystem. Some bacteria specialize in the utilization of lactic or succinic acid. A notable member of the lactate-utilizing guild is *Megasphaera elsdenii*, which converts lactic acid to propionic acid. Lactate and succinate fermentation to propionate is another essential function of the rumen microbiota because the propionate is absorbed and converted to glucose by the host (2–4). It is now known that *M. elsdenii* is also involved in the bioconversion of dietary fats, including production of conjugated linoleic acid (5, 6). However, *Anerovibrio lipolytica* and other bacteria are the major lipolytic species (7).

Protein catalysis by rumen bacteria can be compared and contrasted with fiber catalysis. Like cellulose or starch, proteins are polymers. Protozoa consume intact proteins in feed particles, but bacteria must depolymerize protein and transport the resulting peptides or amino acids. Like all organisms, some form of nitrogen is required for anabolism by bacteria, and many rumen microorganisms express proteinases to this end. *S. bovis* is proteolytic and the growth rate is fastest when free amino acids are available, even though it can also assimilate ammonia (8).

**Figure 1:**
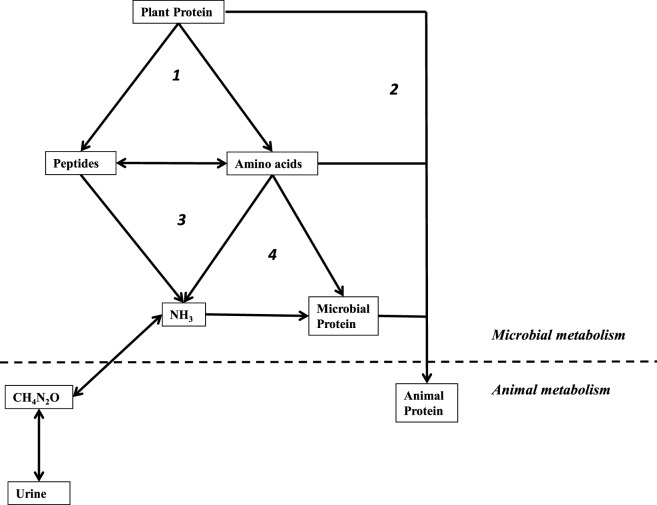
Simplified schematic of amino-nitrogen cycling the rumen. Processes are labeled: (1) proteolysis by microorganisms, (2) “by-pass” protein not deconstructed in the rumen, (3) deamination by microorganisms, and (4) assimilation by microorganisms for anabolic purposes. Used with permission (84).

There are also rumen microorganisms that catabolize amino acids for energy (Figure 1). Protozoa and some Gram-negative bacteria, like *M. elsdenii*, can utilize amino acids and produce ammonia. However, a particular group of rumen bacteria are known for exceptional rates of ammonia production *via* fermentation (i.e., deamination of peptides and free amino acids). This guild of amino acid-fermenters is termed the hyper-ammonia-producing bacteria, HAP or HAB (9). Some of the best-studied HAB are non-proteolytic. Like the saccharolytic bacteria, which metabolize sugars only after other bacteria degrade the polymer, many of the HAB ferment amino acids only after other members of the microbial community depolymerize protein (10). A major difference between catalysis of fiber and protein is that the ruminant host does not need the rumen microflora to utilize protein. Feed protein that is not depolymerized and deaminated is digested in the abomasum and absorbed in the small intestine. Such feed protein that escapes ruminal degradation has been termed *bypass protein*, and it is associated with increased weight gain and feed efficiency (11). Some of the ammonia produced by HAB and other microorganisms is assimilated into microbial protein that can be digested by the host. Excess ammonia is lost.

Certain characteristics are true of all fermentations regardless of the substrate. All fermentations have optimal temperatures, water availabilities, and pH values. The ruminant host helps to meet these needs for its fermentative rumen microbiota. Another requirement of all fermentation is one or more terminal electron acceptors so that reducing equivalents, *i.e*., NAD^+^ and NADP^+^, can be recovered (12). Hydrogen gas (H_2_) is one of the most important terminal electron acceptors in rumen bacteria. An example of an H_2_-producting pathway is the so called *clostridial fermentation*, in which ATP is generated by acetate- or butyrate-kinases and NAD^+^ is regenerated with hydrogenases (12). Bacterial hydrogenases are notoriously subject to end-product inhibition, but the H_2_ is removed by a cross-feeding mechanism that was termed interspecies hydrogen transfer (3, 13). The hydrogen transfer is actually inter-domain because the H_2_-utilizing guild is composed of methanogenic *Archaea*. These methanogens convert H_2_ and CO_2_ into CH_4_. Other methanogens produce CH_4_ from acetate. Eructation of the gases is the hosts’ role in recovery of reducing equivalents (1). Like ammonia production, some methane production is necessary for rumen microbial ecology, but both products represent matter lost from the system.

## Rumen Optimization Hypothesis and Antimicrobial Growth Promoters

When we acknowledge that the microbial activity in the rumen constitutes a natural fermentation, we engender the hypothesis that it can be optimized like any industrial fermentation (3). Applied microbiology has been used since the mid-twentieth century to improve ruminant health and productivity *via* manipulation of the rumen fermentation. As mentioned above, adding starch can decrease the pH of the rumen. Conversely, pH can be increased by adding buffers to the diet (2). Because the rumen is a complex, polymicrobial fermentation, it can also be influenced through the use of antimicrobials. Antimicrobial growth promoters that are administered as feed additives are among the greatest successes to date (14).

Consider the effects of antimicrobial growth promoters in terms of *selective inhibition* and *compensatory product formation*. Selective inhibition is inhibition of specific physiologies. Compensatory product formation is a change in the amount or composition of metabolic products due to selective inhibition. Compensatory product formation can occur in pure cultures as a result of altered physiology. An example outside of the rumen is the cellulolytic bacterium *Clostridium thermocellum*, which converts cellulose into acetate, formate, lactate, ethanol, and H_2_ (15). When the hydrogenases are inhibited by methyl viologen, ethanol, produced by dehydrogenases as an alternative route of NAD^+^ recovery, increases (16). In the case of a pure culture of *C. thermocellum*, it is only the physiology of the culture that is affected. However, the effects of selective inhibition can be ecological as well as physiological. Another example apart from the rumen is industrial ethanol production by yeast. *Saccharomyces cerevisiae* produces the ethanol, but lactic acid bacteria on the feedstock can also produce lactate. Adding an antibiotic, such as virginiamycin, selectively inhibits the growth of lactic acid bacteria, and ethanol production is enhanced. This can be considered an ecological effect because diversity within the fermenter decreases. The rumen and other gastrointestinal habitats are characterized by rapid and continuous flow of matter through the system. Selective inhibition of a metabolic pathway can cause a rapid change in the fitness of the affected organisms. Thus, physiological- and ecological-selective inhibitions are effectively synonymous in the case of the rumen.

The points for optimization of rumen fermentation are end products that exit the rumen. Products leaving the rumen can be divided into two categories according to the usefulness to the host, waste products, and nutrients. Waste products are potential targets for selective inhibition and nutrients are the desired end products of compensatory product formation. An antimicrobial that has a favorable impact on rumen fermentation should selectively inhibit the waste products and compensate in nutrient production. The most widely used and most thoroughly studied ruminant antibiotic growth promoter is the polyether antibiotic, monensin. Ionophores, such as monensin, selectively inhibit members of several guilds of microorganisms and metabolic processes that they carry out (17).

Monensin is known in the ruminant industries as a coccidiostat, but it is also an inhibitor of methanogenesis (18). The gas eructated by a ruminant is composed of waste products, CO_2_ and CH_4_. While these are necessary end products of the rumen fermentation, they also represent mass lost from the system, and are obvious targets for optimization. Additionally, CO_2_ and CH_4_ are greenhouse gases, and the US Environmental Protection Agency indicates that CH_4_ has a global warming potential as much as 36 times greater than CO_2_ over a 100-year period (19). Including monensin in the diet decreases CH_4_ production by as much as 25% (20). Methanogens vary in their sensitivity to monensin (18), and one well-studied species, *Methanobrevibacter ruminantium*, is not sensitive (21). However, CH_4_ production can also be decreased by inhibition of monensin-sensitive H_2_-producing bacteria and protozoa because less H_2_ is available for interspecies hydrogen transfer (17, 18).

Early research identified that monensin and other ionophores increased the ratio of propionic to acetic acid (22). All of the major VFA and amino acids can serve as energy sources, but propionate is the most rapidly utilized by the liver for either oxidation or gluconeogenesis (4). Lactate and succinate are the substrates for propionate production, and both metabolic pathways involve dehydrogenases and the reduction of reducing equivalents (12). Thus, propionate is an alternative electron sink and a compensatory product of CH_4_ inhibition. It has been proposed that the shift from a methanogenic to a propionic electron sink is governed by the sensitivity of rumen methanogens to acidic pH, which would explain the shift in acetate: propionate when cattle are switched from a forage-based to a grain concentrate-based diet (22). The pH-based explanation of compensatory propionate production is consistent with the mechanism of action of ionophores (described below). However, it is also important to note that known propionate-producing bacteria, such as *M. elsdenii* and *Selenomonas ruminantium*, are members of Class *Negativicutes*, known for their outer membranes (23). The outer membrane of these Gram-negative species confers insensitivity to ionophores (24). We would expect ionophores to select for these propionate-producers even if reducing equivalent disposal were not considered.

Nitrogenous waste is another target for selective inhibition (Figure 1). It has long been recognized that ionophores also inhibit rumen amino acid degradation (18). However, prior to the discovery of the HAB, all known amino acid-fermenting bacteria (e.g., *M. elsdenii*) were Gram-negative and ionophore-insensitive (9, 24). Most of the characterized HAB are members of Order *Clostridiales* with classical, Gram-positive cell envelopes that render them susceptible to ionophores (9, 25–28). The ciliates, the other major ammonia producers, are also inhibited, but there is evidence that they adapt to monensin (29). When HAB and other ammonia producers are inhibited, the rate of free amino acid and peptide catabolism is decreased, and more amino-nitrogen is available for the host to absorb in the small intestine (9). As previously mentioned, protein, peptides, and amino acids that escape rumen degradation have been called *bypass protein*, and are associated with increased weight gain and feed efficiency.

Antibiotic growth promoters have been very important tools in ruminant production for decades. In 1989, Russell and Strobel (17) estimated that ionophores alone were responsible for a feed savings of 560,000,000 USD. A more recent estimate by Capper and Hayes (30) indicates abolishing antibiotics and other growth promoting technologies would increase production costs by 9.1%. Moreover, they and others point to the environmental benefits (i.e., decreased carbon and nitrogen emissions) when growth promoters are used (18, 30). However, a considerable body of evidence now indicates that growth promoting and veterinary uses of antibiotics contributes to antibiotic-resistant bacteria in food animals (31–33). These concerns are compounded by the spread of antibiotic-resistant food borne pathogens and the presence of antibiotic residues in compost and fertilizer from animal operations (34, 35). Clearly, it is in our best interest to minimize the use of clinically important antimicrobials while maintaining, or even expanding, the benefits of growth promoting technologies.

**Figure 2:**
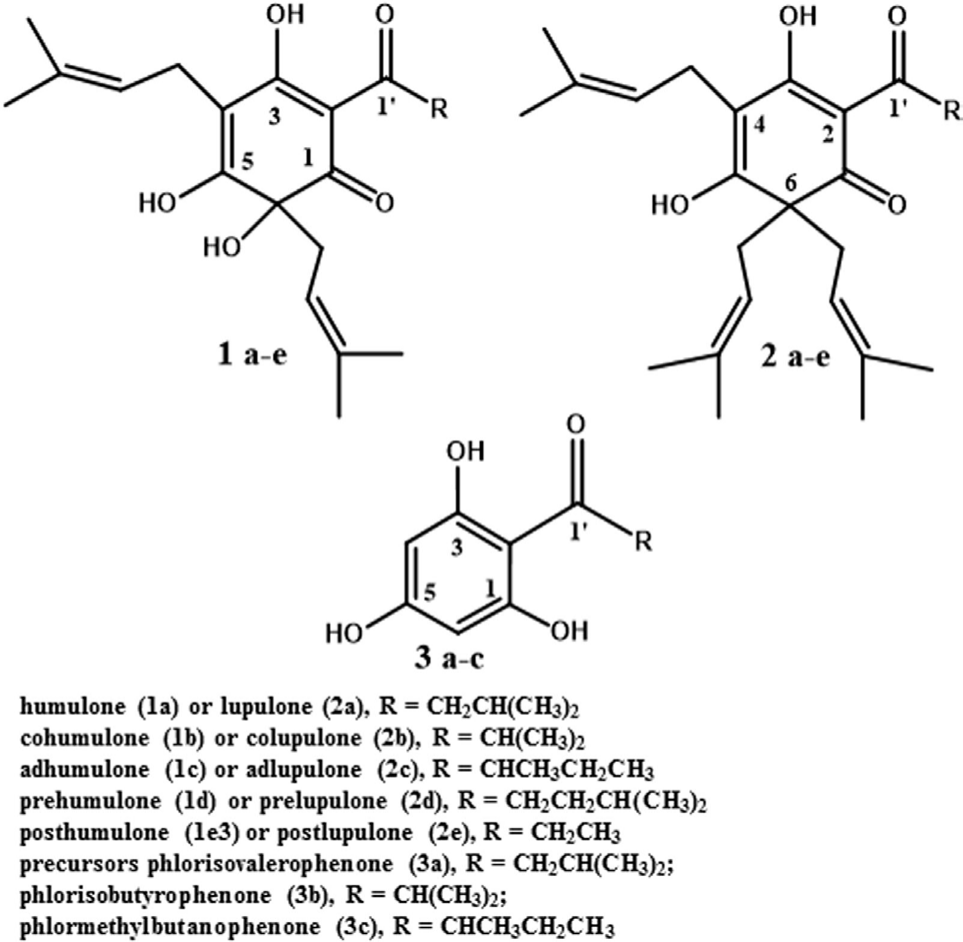
Structures of hops bitter acids and biosynthetic precursors.

Researchers have proposed a variety of alternative antimicrobials as ruminant growth promoters. The candidate compound should have an antimicrobial mechanism of action dissimilar to clinically important antibiotics. Ionophores fit the mechanism of action criterion, and there is evidence that ionophore-resistant bacteria are not typically resistant to other classes of antibiotics (36). A study by Simjee and co-workers (37) also indicated that monensin-resistance is not highly heritable. However, the acute toxicity of monensin to humans and horses makes it a perennial concern (36, 38). The purpose of this review is to consider secondary metabolites from the hops plant (*Humulus lupulus* L.), as feed antimicrobial growth promoters. A variety of rumen-active phytochemicals have been considered as feed additives, and many have merits (39). We believe that hops secondary metabolites, particularly the beta-acids (lupulone and its derivatives; see Figure 2, structure 2a–e), have a special role as model rumen-active phytochemicals. The basis for this assertion is the considerable body of literature available on the plant and compounds and the spectrum of activity and mechanism of action, which are similar to feed ionophores. Information on hops essential oils and prenylated flavonoids is provided as well in the next section to illustrate the diversity present in the plant.

## Taxonomy and Distribution of Hops

*Humulus lupulus* L. is a member of the family Cannabaceae, which also contains the genus *Cannabis* (40). It is a dioecious vine, with male and female flowers growing on separate plants (40, 41). Hops is indigenous to the Northern hemisphere, although it is grown in both hemispheres (42). The species variety native to Europe is *H. lupulus* var. *lupulus*, and four other species varieties have been described based on native distribution and morphology:*H. lupulus* var. *cordifolius* (Miquel) Maximowicz, a native of Japan and possibly of parts of mainland Asia; *H. lupulus* var. *neomexicanus* Nelson and Cockerell (native to western North America); *H. lupulus* var. *lupuloides* E. Small (native to eastern and central North America); and *H. lupulus* var. *pubescens* E. Small (native to the midwestern US) (42). Some commercial cultivars are the results of crosses between native plants from different continents (42), and some wild populations may be the result of introducing plants into an area and letting them grow wild (43). Wild populations in various regions have been studied because of their potential value as a source of germplasm for commercial hops cultivation (44–46), or because comparisons of morphological or chemical traits can provide information on relationships among populations (47, 48).

## Localization and Accumulation of Hops Secondary Metabolites

**Figure 3:**
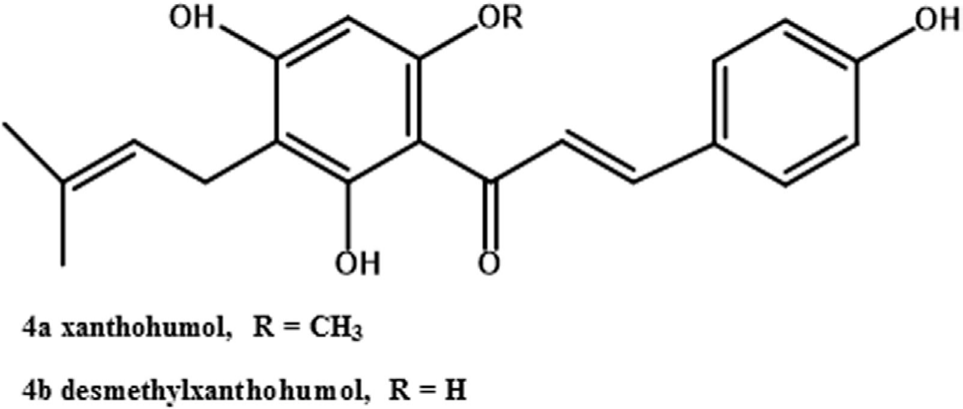
Structures of hops prenylated flavonoids.

Only the mature female inflorescences (cones) of hops are used in the beer brewing industry (40). The female cones, which develop over a few weeks after flowering, consist of clusters of bracts subtended by bracteoles, all grouped around a central axis (49), also referred to as a central rachis (50). On these bracts and bracteoles are glandular trichomes (51), which have been described both as distinct from lupulin glands (52) and as including both lupulin glands (also called peltate trichomes) and the smaller bulbous trichomes (53). Trichomes are present on leaves as well (53). They contain secondary metabolites such as prenylated flavonoids (54), essential oils (mono- and sesquiterpenes; 53), and bitter acids (55). This latter class of secondary metabolites includes the α-acids (humulone and derivatives, Figure 2, structures *1a–e*) and the β-acids (lupulone and derivatives, Figure 2, structures *2a–e*). Bitter acids and prenylated flavonoids have been found at lower concentrations in male than in female inflorescences (56). Some of the secondary metabolites of hops are found in other tissues of female plants besides trichomes. A couple of sesquiterpenes are found in leaves and flowers (51), and prenylated flavonoids xanthohumol and desmethylxanthohumol (Figure 3, structures *4a* and *4b*) are at low concentrations in leaves and immature cones (55). Lipophilic compounds like mono- and sesquiterpenes (51), and bitter acids and prenylated flavonoids (55), are most abundant in trichomes.

The changing concentrations of prenylated flavonoids, essential oils, and bitter acids in mature trichomes indicate that hops secondary metabolite content is closely related to trichome maturity. Extraction of bitter acids and essential oils from hop cones over a few weeks revealed that both types of compounds increased during this period (52). Extraction of bitter acids from individual trichomes revealed a similar trend of increased bitter acid concentration with increasing maturity (57). In those studies, electron microscopy of trichomes has demonstrated that while trichomes are initially concave, they gradually fill out as bitter acid concentrations increase (52, 57), and the alpha-acid content of trichomes is positively correlated with trichome volume (50).

## Biosynthesis of Bitter Acids and Prenylated Flavonoids

The genes, enzymes, and intermediates of hop bitter acid and prenylated flavonoid biosynthesis have been studied extensively, due to interest in manipulating their production for brewing purposes. Precursors of the moieties comprising bitter acids, and the associated enzymes that have been identified, are listed in Table 1. Some of the earlier studies (58) demonstrated that feeding ^14^C-labeled acetic acid to cone-bearing hops plants led to isolation of ^14^C-labeled humulone, lupulone, and colupulone (Figure 2, structures *1a, 2a*, and *2b*, respectively), indicating an acetate precursor. Because acetate can be converted into acetyl-CoA, which can react with carbon dioxide to form malonyl-CoA, a precursor of some aromatic compounds (59), a role for acetate agrees with later findings (60) that the 6-carbon ring and acyl side chain at C-2 (Figure 2, structures *1* and *2*) are formed through the biosynthesis of an acylphloroglucinol nucleus (Figure 2, structures *3a* to *3c*). A crude enzyme extract from flowers or cones, incubated with malonyl-CoA (likely precursor of the 6-carbon ring) and isovaleryl-CoA or isobutyryl-CoA (likely precursors of the acyl side chain at C-2 in Figure 2), catalyzed the reactions producing the acylphloroglucinol compounds phlorisovalerophenone (Figure 2, structure *3a*), and phlorisobutyrophenone (Figure 2, structure *3b*) (60). Structure *3a* is a likely precursor of lupulone and humulone (Figure 2, structures *2a* and *1a*, respectively), and structure *3b* is a likely precursor of colupulone and cohumulone (Figure 2, structures *2b* and *1b*, respectively) (60). The purification of the biosynthetic enzyme, phlorisovalerophenone synthase (VPS), from trichomes has been described (61), as have the cloning of the gene and its trichome-specific expression (62). *In situ* hybridization of *VPS* RNA in trichomes demonstrated that *VPS* gene expression occurs only during a late stage of trichome development (53), supporting the relationships between bitter acid accumulation and trichome maturity described above.

**Table 1:** Biosynthetic precursors and enzymes confirmed for lupulone or humulone and their derivatives.

Moiety	Precursor	Enzyme(s) involved	Reference
6-carbon ring (structures *1* to *3*, Figure 2)	Malonyl-CoA	VPS (valerophenone synthase)	(61, 62)
C-2 acyl side chain to structure *3* (Figure 2)	Isovaleryl-, 2-methylbutyryl, or isobutyryl-CoA	HlCCL2 and HlCCL4 (carboxyl-CoA ligases)	(55)
Alkyl moiety on C-2 acyl side chain of humulone (structure *1a*, Figure 2)	Leucine	Enzymes not characterized in refs. 64 or 66; BCAT1 (branched-chain amino transferase) proposed to convert leucine into a precursor of isovaleryl-CoA	(64–66)
Prenyl group on C-4 of structures *1* to *3* (Figure 2)	Deoxyxylulose-5-phosphate in plastidial isoprenoid pathway	Enzymes not characterized in ref. 66; HlPT1 (prenyltransferase) prenylates C-4	(63, 66, 70)
Prenyl group on C-6 of humulone	Deoxyxylulose-5-phosphate in plastidial isoprenoid pathway	Enzymes not characterized in ref. 66; HlPT2 (prenyltransferase) prenylates C-6	(63, 66, 71)
Oxygen on C-6 in humulone and cohumulone (Figure 2, structures *1a* and *1b*)	Molecular oxygen	Oxygenase; enzyme not characterized	(72)

*These have not been confirmed for all the bitter acids listed in Figure 2*.

Work has been done to determine the precursors of the acyl side chain at C-2 of α- and β-acids. The alkyl moiety at C-1′ (the R group in structures *1* to *3* of Figure 2) appears to be derived from the carbon backbone of aliphatic amino acids, and feeding studies with ^14^C-labeled leucine and isoleucine led to incorporation of isoleucine into 2-methylbutyrate (a likely precursor of compounds *1c* and *2c*), and of leucine into isovalerate and lupulone (64). Gene expression studies revealed that among the genes expressed strongly in hop trichomes were those encoding branched-chain aminotransferase enzymes for the biosynthesis and catabolism of branched-chain amino acids (leucine, valine, and isoleucine) (65). The trichomes were also the tissues highest in isovaleryl-, isobutyryl-, and 2-methylbutyryl-CoA, which are derived from the breakdown products of leucine, valine, and isoleucine, respectively (65). Xu et al. (55) identified and cloned some hops carboxyl-CoA ligase genes, including two (*HlCCL2* and *HlCCL4*) that were expressed most strongly in mature cones or trichomes. They encoded enzymes catalyzing conversion of metabolites of isovalerate, isobutyrate, and 2-methylbutyrate into their corresponding CoA esters (55). When *HlCCL2* was co-expressed in yeast with a *VPS* gene, structure *3a* was produced (Figure 2), and coexpression of *VPS* and *HlCCL4* produced structures *3b* and *3c* (Figure 2) (55). Structure *3c* is a probable precursor of adlupulone and adhumulone (Figure 2, structures *2c* and *1c*, respectively).

The prenyl side chains at carbons 4 and 6 of humulone (Figure 2, structure *1*) is synthesized from glucose *via* the plastidial isoprenoid pathway (66), in which deoxyxylulose-5-phosphate is the precursor of the isopentenyl-pyrophosphate (IPP) or dimethylallylpyrophosphate (DMAPP) building block of isoprenoids (67). The biosynthetic origin of lupulone prenyl side chains does not appear to have been determined. In agreement with the humulone labeling and NMR studies, expressed sequence tags (ESTs) of the plastidial isoprenoid pathway were found in cDNA libraries from hops trichomes (51, 54), but few ESTs (54) or none (51) were present from the cytosolic isoprenoid pathway, which is characterized by a mevalonate precursor to DMAPP and IPP (67).

The prenyl side chains are added to bitter acids by prenyltransferases. Incubation of structures *3a* and *3b* with DMAPP and a crude enzyme extract from hops trichomes led to formation of mono- and diprenylated versions of structures *3a* and *3b* (63), confirming the presence of prenyltransferase enzyme activity in the trichomes. Incubating this crude extract with deoxyhumulone (Figure 2, structure *1a* minus the -OH group at C-6) led to some production of humulone, indicating that deoxyhumulone might be a precursor (68). A prenyltransferase gene (*HlPT1*, catalyzing the transfer of a prenyl group to an aromatic nucleus) was identified in a hops trichome cDNA library and found to be expressed most strongly in the trichomes of young cones (69). Assays of the expressed prenyltransferase revealed that it was capable of only one prenylation step, namely the addition of the prenyl group at C-4 of the structures in Figure 2 (70). Therefore, additional enzymes are needed to complete the biosynthesis of α-acids (two prenyl groups) and β-acids (three prenyl groups) (70). A similar gene (*HlPT1L*), as well as an additional gene (*HlPT2*, encoding the prenyltransferase catalyzing transfer of the additional prenyl groups to alpha- or β-acids), were cloned from hops trichomes (71). When both genes were expressed in yeast, along with the *HlCCL2, HlCCL4*, and *VPS* genes, β-acids were produced, as well as various other prenylated acylphloroglucinols (71). No α-acids were produced in this yeast expression system, indicating that additional enzymes were needed to convert deoxyhumulone or related compounds into humulone and its derivatives (71). A late-stage hydroxylation of C-6 of structure *1* is supported by the determination that ^18^O_2_, when fed to whole hop plants, is incorporated only into the oxygen atom bonded to C-6 of humulone and cohumulone (72).

Biosynthesis of xanthohumol (Figure 3, structure *4a*), the most abundant prenylated flavonoid in hops (73), involves both the phenylpropanoid and isoprenoid biosynthetic pathways. Many phenylpropanoid biosynthetic genes were present in a hop trichome cDNA library (54). A chalcone synthase cDNA from hop cones was incubated with *p*-coumaroyl CoA and produced naringenin (74). A prenyltransferase capable of prenylating acylphloroglucinols was also capable of prenylating naringenin chalcone (the precursor of naringenin), producing desmethylxanthohumol (Figure 3, structure *4b*) (70). Methylation of desmethylxanthohumol to produce xanthohumol was achieved in the presence of an *O*-methyltransferase cloned from hop trichomes (54).

## Factors Affecting Concentrations of Some Hops Secondary Metabolites

Concentrations of bitter acids (44, 56, 75), prenylated flavonoids (56), and essential oils (44) can vary within cultivars or populations from year to year. The year-to-year variation may be due to differences in temperature or precipitation because De Keukeleire et al. (56) observed generally higher bitter acid concentrations in a year with a wet summer, and lower concentrations in a year with a hot summer. Despite environmentally influenced fluctuations, secondary metabolite concentrations tend to stay within certain ranges for a given genotype, indicating genetic as well as environmental effects. Cultivated hops are categorized in the brewing industry by their bitter acid and essential oil content. Aroma hops contain a maximum of 5–7% w/w α-acids and <1% w/w essential oil, while bitter hops contain 7–10% w/w α-acids and 1–2% w/w essential oil, and high-α hops contain over 10% w/w bitter acids and 1.5–3% w/w essential oil (75). The ratios of α- to β-acids in these cultivar classes tend to increase in that same order (<1–2 for aroma hops, 2 for bitter hops, and 2–3 for high-alpha hops) (75). Wild populations of hops tend to have bitter acid concentrations on the order of that observed for aroma or bitter hops (46). For example, in a survey of 22 wild Italian hops populations, the α-acid content was 1.7–7.3%, and the β-acid content was 1.2–3.9%, with only two populations having an α- to β-acid ratio greater than 2 (46). In a survey of wild hops populations from the Czech Republic, Switzerland, France, and Russia, the α- to β-acid ratio was below 1.5 for all (44).

Because secondary metabolite production is partly under genetic control, types and concentrations can also help to identify geographic origins. Wild European and North American hops can be distinguished from each other by their relative amounts of certain essential oils and bitter acids (45). Wild hops from the southwestern United States differ from other North American hops in lacking several 4′-*O*-methylchalcones structurally similar to xanthohumol, but with different placement or number of methyl groups (48). Types and amounts of secondary metabolites can also serve as a fingerprint to identify individual cultivars. Concentrations of essential oils (76, 77) or non-prenylated flavonoids (76) have been used for this purpose. A dichotomous key to seven Czech cultivars was constructed based on concentration or presence of selected essential oils, flavonoids, and other phenolic compounds (78). Another study of Czech hops cultivars (79) classified them according to bitter acid, prenylated flavonoid, total phenolic and flavonoid, essential oil, antioxidant, and proteinase inhibitory activities. This type of clustering based on multiple parameters may help to identify cultivars with qualities relevant to other industries besides brewing.

## Hops: A Source of “Phyto-Ionophores”

It has long been recognized that the antimicrobial activity of hop secondary metabolites could have value as clinical antiseptics (80). However, the mechanism of action was first elucidated by Teuber and Schmalreck in the 1970s (81). They noted that the most hydrophobic hop resin components were also the most antimicrobial, and hypothesized that those components must be active against the bacterial cell membrane. *Bacillus subtilis* cells were treated with lupulone (a β-acid; Figure 2, structure 2a), humulone (an α-acid; Figure 2, structure 1a), isohumulone or humulinic acid fractions of hop resins. The secondary metabolites inhibited growth and prevented the transport of radioactively labeled α-methyl-d-glucopyranoside (a sugar) and a variety of amino acids. Those results alone could be the result of membrane leakage or other action on bioenergetics that would stop energy-dependent transport. However, lupulone also caused serine efflux from *B. subtilis* membrane vesicles that were loaded with ^14^C-serine, which was indicative of membrane leakage.

Some ionophores are highly specific in terms of ion selectivity. Protonophores, such as 3,3′,4′,5-tetrachlorosalicylanilide, transport only protons. Monensin is less selective, but it is generally considered a proton/potassium antiporter because those are the two major monovalent cations in disequilibrium across an energized cell membrane (17). Membrane perturbation induced by hop constituents is even less specific than monensin, but the effects of hop secondary metabolites and feed ionophores were similar enough to warrant comparison.

## A Relevant Spectrum of Activity

**Figure 4:**
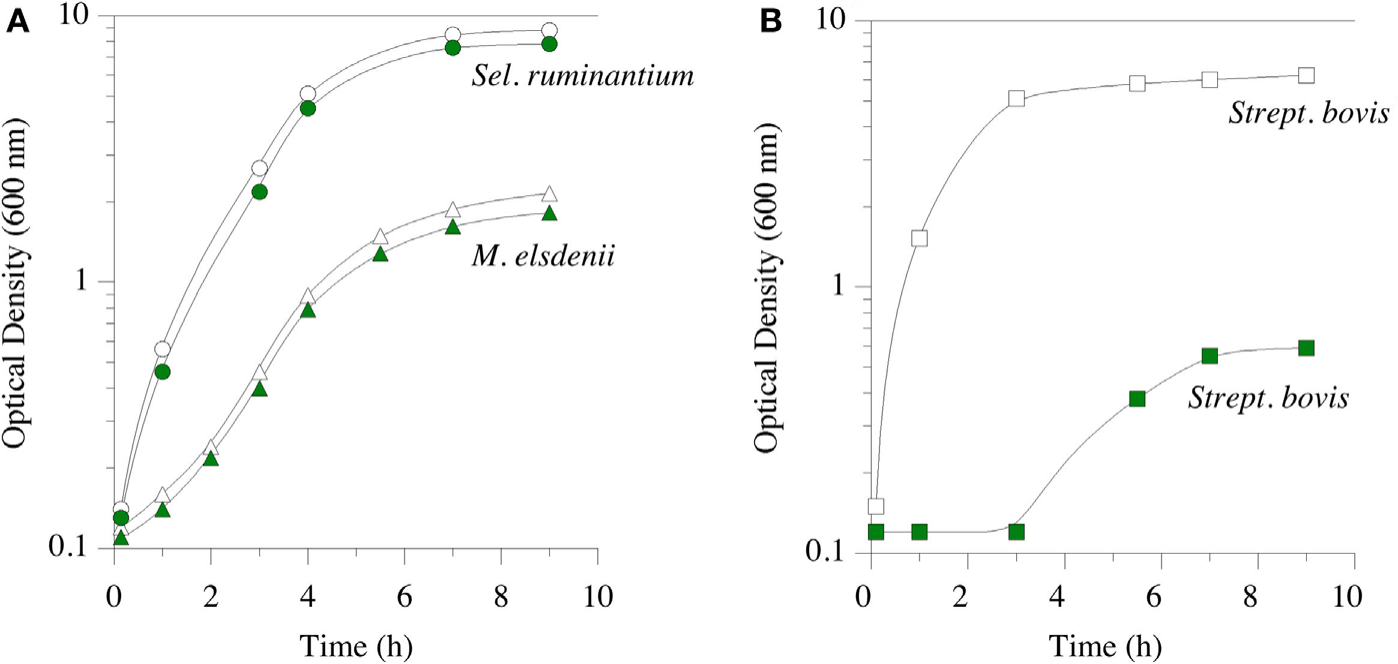
Growth of *Selenomonas ruminantium* (circles) and *Megasphaera elsdenii* (triangles) in part **(A)**, and *Streptococcus bovis* (squares) in part **(B)**. Green symbols indicate hops extract (30 ppm β-acid) was added prior to inoculation. Data adapted from Ref. (83).

Feed ionophores and hop bitter acids have similar spectra of activity; i.e., they inhibit the same microorganisms (17, 81). They both inhibit bacteria with a classical Gram-positive cell envelope. *B. subtilis*, which Teuber and Schmalreck employed to determine the mechanism action, is a long-standing Gram-positive model. Hops β-acid, purportedly lupulone, also inhibits important Gram-positive pathogens, such as *C. perfringens* (82). As mentioned above, HAB and other Gram-positive rumen bacteria are sensitive to monensin, but Gram-negative rumen species are not. The Gram-positive spectrum of activity for a mixture of β-acids, consisting of compounds 2a through 2c (Figure 2) was shown in pure culture growth experiments (Figure 4). The Gram-positive, *S. bovis* was inhibited by β-acids, as it is by monensin (83). When an inhibitor suppresses Gram-positive bacteria, like *S. bovis*, lactic acid production is limited. Decreasing lactate production can ameliorate lactic rumen acidosis (2). *S. ruminantium* and *M. elsdenii* belong to Class *Negativicutes*, and have a Gram-negative cell envelope. The growth of these two bacteria was not inhibited by β-acids [Figure 4; (83)]. The propionate-producing *Negativicutes*, like *M. elsdenii*, are not sensitive to monensin, and it is thought that this resistance to ecological perturbation by feed ionophores is responsible for the increased proportion of propionate when ionophores are fed. β-acids, like ionophores, decreased acetate production by washed cell suspensions of uncultivated, mixed rumen microorganisms (hereafter called *washed cell suspensions*) without decreasing propionate (83).

**Figure 5:**
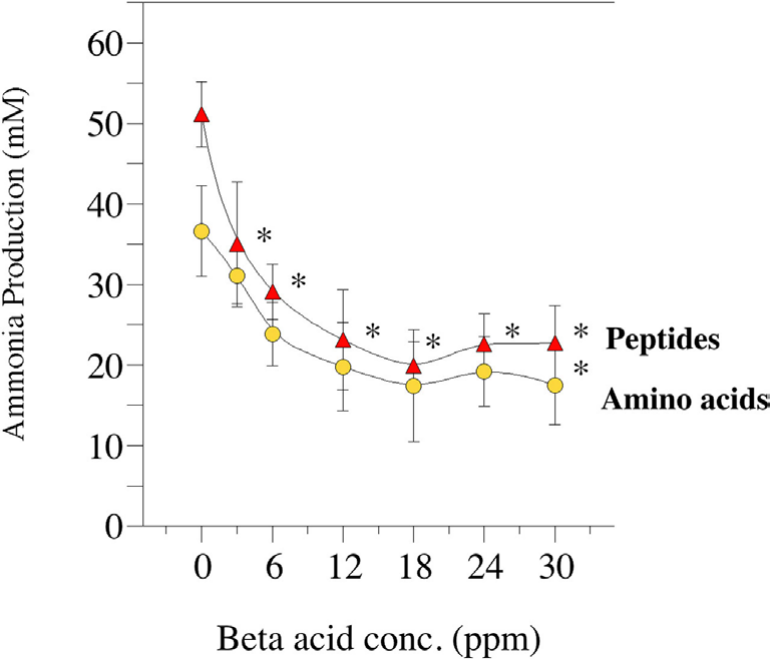
Effect of hops extract, containing 45% beta-acids (a mixture of lupulone, colupulone, and adlupulone) on ammonia production by washed (uncultivated) cell suspensions from the goat rumen. Ammonia production from peptides (triangles) or amino acids (circles) after 24 h incubation (39°C) are shown. Error bars indicate SEM. Asterisks indicate treatments that are different than the 0 ppm control. Data adapted from Ref. (84).

**Figure 6:**
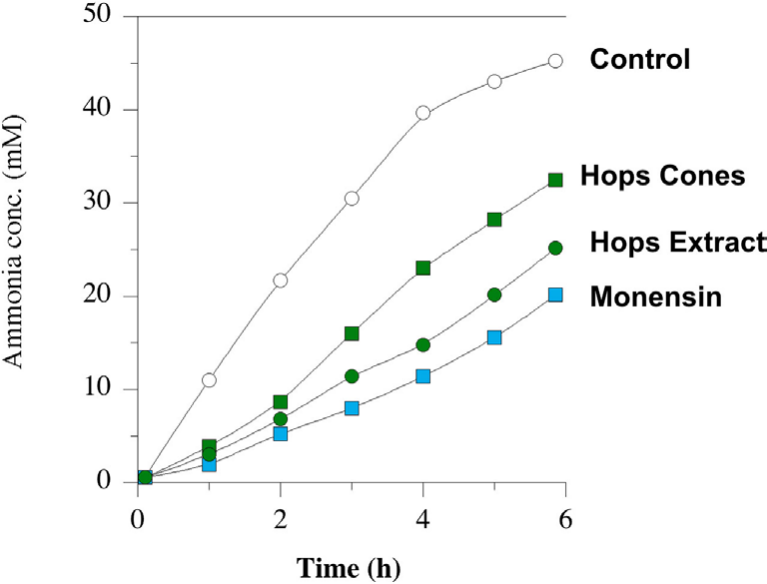
Ammonia production by mixed rumen microbes. Washed cell suspensions were incubated with peptides and amino acids. No addition (open circles), 10 µmol l^–1^ monensin (blue squares), 2% w/v dried hops cones (green squares), and hops extract (60 ppm beta-acid) (green circles). Data adapted from Ref. (85).

Amino acid degradation can also be evaluated using washed cell suspensions. Figure 5 shows the effect of β-acid concentration on ammonia production from either free amino acids or peptides (84). Figure 6, on the other hand, compares inhibition of ammonia production by a β-acid-rich extract to unprocessed hops cones and monensin (85). It is important to note that the substrates in these experiments were free amino acids and peptides, not protein. However, Lavrenčič and colleagues (86) used similar *in vitro* assays to show that two different hops varieties could also decrease proteolysis by rumen microorganisms, which could include proteolytic bacteria or ciliate protozoa. Hops secondary metabolites, like feed ionophores, inhibit both the proteolysis and amino acid fermentation stages of rumen protein degradation.

The advantage of washed cell suspension and similar *in vitro* fermentations is that the microorganisms can include the full diversity that is in the rumen, not just a few laboratory models. However, absorption by the host does not confound the measurements. Thus, washed cell suspensions show the net metabolic outputs of the rumen microbial community. Van Nevel and Demeyer first discovered the effects of monensin on rumen microbiology using *in vitro* mixed rumen microorganisms (18).

Three varieties of hops cones have also been tested in a continuous *in vitro* rumen fermentation system (87, 88). Like washed cell suspensions, this system starts with uncultivated rumen microorganisms, but continuous systems allow adaptation over time. In this case, Gram-positive bacteria, such as *S. bovis*, were inhibited and the proportion of propionate to acetate increased. The number of methanogens and methane production decreased in the presence of hops, which would be an expected result with feed ionophores. Indeed, similar results were observed when monensin was used in the same fermentation system (88).

We can see that the spectrum of activity of hop β-acids is like that of feed ionophores because: (1) Gram-positive rumen bacteria are inhibited, (2) Gram-negative rumen bacteria are insensitive, (3) the shift in fermentation acid production is like ionophores, and (4) proteolysis, ammonia production, and methane production decrease.

## Relationship to pH and Impact on Transmembrane Gradients

**Figure 7:**
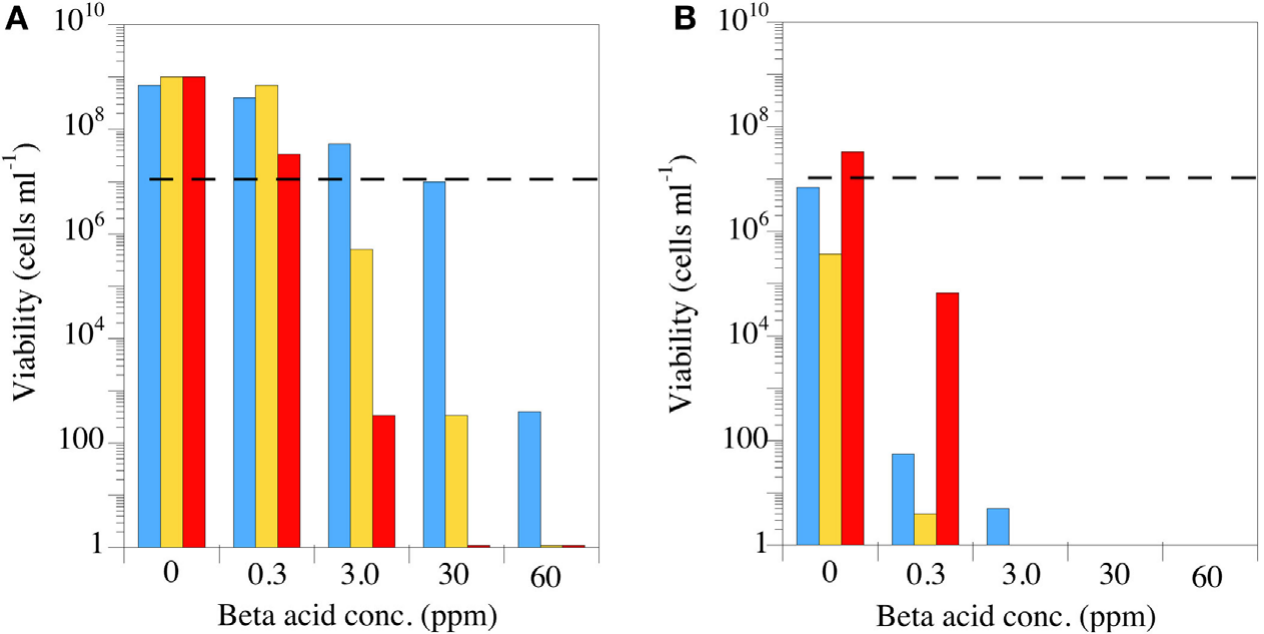
The effect of hops β-acids on the growth and survival of Peptostreptococcus anaerobius (blue bars), *Clostridium sticklandii* (gold bars), or *Clostridium aminophilum* (red bars). The incubations (24 h) were carried out at pH 6.7 **(A)** or pH 5.6 **(B)**, and the dashed lines indicate initial viable cell number. Extract composition is as described in Figure 5. Data adapted from Ref. (85).

The most important feature for a putative antimicrobial rumen modifier is the spectrum of activity, but the spectrum is largely dictated by the mechanism of action. Based on the early work with *B. subtilis*, it was a reasonable hypothesis that β-acids would disrupt the membrane integrity of Gram-positive rumen bacteria (85). A key feature of a proton-transporting ionophore’s effect on cell membranes is that the ionophore becomes more potent as the pH decreases (89). There are several ways to determine increased efficacy at acidic pH including lower minimum inhibitory concentrations or steeper time-kill curves. Figure 7 simply shows the effect of increasing concentrations of the previously mentioned β-acid mixture on the viable number of three HAB pure cultures at neutral and acidic pH [Figure 7; (85)]. These three Gram-positive bacteria were all sensitive at neutral pH at β-acid concentrations between 3 and 30 ppm. Decreasing the pH from 6.7 to 5.6 decreased the viable numbers in all cases. In some cases, pH made the difference between a bacteriostatic effect, in which the bacteria simply do not grow, and a bactericidal effect, in which the cells are killed.

**Figure 8:**
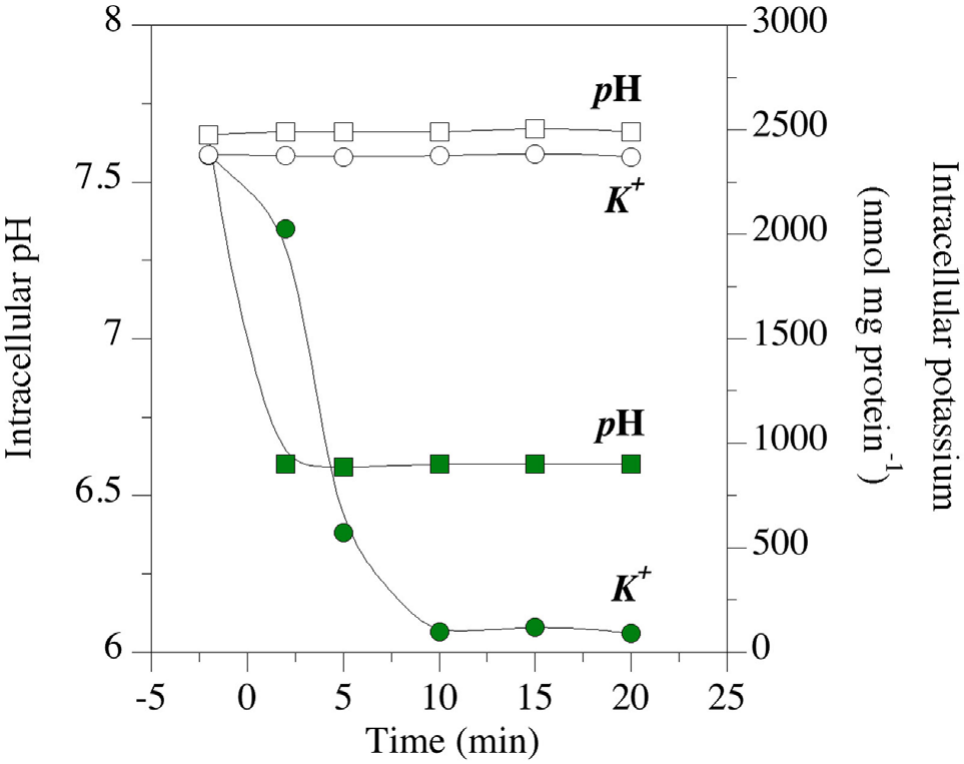
Effect of hops β-acids on transmembrane monovalent cation gradients of the HAB, *Clostridium sticklandii*. The intracellular pH (squares) and intracellular potassium (circles) of energized cell suspensions. Open symbols are controls. Green symbols indicate suspensions to which β-acids were added at 0 min. Data adapted from Ref. (85).

The effects of a putative ionophore on membrane bioenergetic parameters can also be measured. The effects of mixed β-acids on intracellular pH and intracellular potassium are shown in Figure 8 (85). The test organism was *Clostridium sticklandii*, and it was maintaining a transmembrane pH (ΔpH) gradient of approximately 1.0 pH units (intracellular pH 7.6). The ΔpH collapsed within 2 min when β-acids were added, and the intracellular pH was equal to the extracellular pH (6.7). The loss of ΔpH interferes with bacterial physiology in two ways. First, ΔpH along with the difference in charge across the membrane (ΔΨ) comprise protonmotive force, which is utilized for transport and the establishment of other gradients (90). When the membrane depolarizes and protonmotive force dissipates, other concentration gradients, such as valuable ATP, must be used for transport. Second, the cytoplasmic pH could fall out of the optimal range for the cell’s enzymes. It is noteworthy that cytoplasmic acidification is independent of the effects of fermentation acids. It was once thought that fermentation acids were metabolic “uncouplers” of protonmotive force, like ionophores. However, it has been shown that intracellular anion accumulation is the primary cause of growth inhibition by fermentation acids (91).

*Clostridium sticklandii* also lost intracellular potassium $\left({\text{K}}_{\text{I}}^{+}\right)$ when β-acids were added [Figure 8; (85)]. Like many growing bacteria, *C. sticklandii* cells maintain a ${\text{K}}_{\text{I}}^{+}$ of more than 2 µmol/mg cell protein. When the cells were treated with β-acids, ${\text{K}}_{\text{I}}^{+}$ was negligible in 10 min. A homeostatic concentration of potassium is necessary for the function of ribosomes, many enzymes and maintenance of the sodium gradient. However, intracellular turgor is the key function of ${\text{K}}_{\text{I}}^{+}$ (92). Potassium maintains water inside the cell, and turgor associated with water (as much as 20 atmospheres in Gram-positive cells) provides the kinetic energy for cytokinesis in cell division. When ${\text{K}}_{\text{I}}^{+}$ is lost, plasmolysis occurs and the cells cannot divide.

The foundational work with *B. subtilis* shows that hop α- and β-acids are less specific than ionophores like monensin because they allow the efflux of larger molecules, like serine (81). However, the impact on the transmembrane gradients of monovalent cations is much like the proton/potassium antiporter, monensin. Furthermore, the hop spectrum of activity against rumen microorganisms impacts rumen microbial ecology in the same way that we expect from feed ionophores.

## Hops and Ruminants *In Vivo*

Few studies have been published on the effects of hops or hop extracts on nutrient digestion/metabolism, or on productive performance of ruminant animals. In a meeting abstract, Schmidt et al. (93) described the effect of β-acids on *in vivo* ruminal fermentation of a concentrate diet containing 90% corn and 10% alfalfa haylage. The study used four ruminally cannulated steers that were supplemented with 0, 16.5, or 33 mg β-acid/kg diet. Intake and methane emission decreased, while ruminal pH and lactic acid concentration increased linearly with β- acids addition. However, the acetate: propionate ratio, and digestibilities of fiber, starch, and protein were not affected by β-acids addition. Additionally, β-acids increased total protozoa and *Entodinium* spp. The results indicated that addition of β-acids at the dietary concentration of 16.5–33 mg/kg diet resulted in more efficient ruminal fermentation and starch digestion. In contrast, Uwituze et al. (94) reported that supplementing 1, 8, 16, 24, and 30 mg β-acids/kg DM to steers fed a diet containing 64.8% corn, 10% alfalfa hay, and 15% dried corn distillers grains had no effect on rumen pH, concentrations of VFA or lactic acid, or on acetic acid to propionic acid ratio. Supplementation of β-acids up to 24 mg/kg DM also did not affect feed intake or total tract digestibilities of DM, OM, protein, starch, or crude fat. These results seem to indicate that addition of hop β-acids up to 30 mg/kg DM had little effect on nutrient digestibility of cattle fed a concentrate diet. In a more recent study, Axman et al. (95) found that adding 25 and 50 mg/kg DM of β-acid extracts to a corn based concentrate diet did not affect heifers’ growth performance (feed intake, growth rate, and feed efficiency), which was similar to that observed when feeding 33 mg/kg DM of monensin. Please note that the trials by Uwituze et al. and Axman et al. were reported in a reputable, but non-peer-reviewed forum.

To the best of our knowledge, Wang and colleagues (96) conducted the only peer-reviewed study evaluating the potential of hops as a feed additive for cattle. In this study, hops were added to a barley-based growing diet at levels of 0, 119, 238, and 476 mg/kg DM, and to the finishing diet at levels of 0, 238, 476, and 952 mg/kg DM. The hops used in this study contained 84 g of β-acids/kg DM, which resulted in dietary concentrations of β-acids up to 40 and 80 mg/kg DM in the growing and finishing diets, respectively. The results showed that inclusion of hops in growing or finishing diets at these rates did not affect the feed intake, growth, feed efficiency, carcass characteristics, or fatty acid composition of diaphragm tissue of steers. However, growth rate of steers supplemented with the highest level of hops during the growing and finishing period was 6% higher than the growth rate of the control group. These results suggest that higher concentrations of hops in the diet may be required to improve feed utilization and growth in feedlot cattle. Further research is needed to evaluate the applicability of hops and hop β-acids extract as a feed additive in the cattle industry.

Table 2 summarizes demonstrated effects of hops or hops compounds on ruminants and rumen metabolism (83–85, 93, 94, 97). Known effects of feed ionophores are also included. There have been many experiments that included ionophores, and the reviews cited here cover three decades. Please see these and other references for a complete review of feed ionophores (14, 17, 98).

**Table 2:** Demonstrated effects of hops and hops bitter acids or ionophores.

Effect	Hops (or hops bitter acid)	Ionophores
**Animal performance**		
Increased average daily gain	Yes (96), no (93)	Yes (14, 17, 22)^a^
Increased gain:feed	No (93, 96)	Yes (14, 17, 98)
Increased carcass weight	No (96)	Yes (14, 17, 98)
**Rumen metabolism**		
Increased pH	Yes (83, 93), No (93, 94)	Yes (14, 17, 18, 83, 98)
Decreased A:P	Yes (83, 96, 97), No (93)	Yes (14, 17, 18, 83, 98)
Decreased ${\text{NH}}_{4}^{+}$	Yes (84, 85, 97)	Yes (14, 17, 18, 98)
Decreased CH_4_	Yes (93, 97)	Yes (14, 17, 18, 98)
**Other benefits**		
Decreased coccidia	Not reported	Yes (14, 17)

*References discussing the effects listed in the first column are given in parentheses, with yes references supporting the increase or decrease following hops or ionophore treatment and no references not supporting the listed increase/decrease*.

*Gain:feed, amount of weight gained relative to the amount of feed given; A:P, acetate-to-propionate ratio; ${\text{NH}}_{4}^{+}$, ammonium or ammonia; CH_4_, methane*.

*^a^References (14, 17, 98) are reviews of ionophore research, rather than discrete studies*.

## Economics of Hops for Ruminants and Future Directions

The hops plant is a high value food ingredient, not an inexpensive feedstock or co-product like those typically fed to animals. The situation is also complicated by the variations of the hops market price (99). For example, the average 5-year cost (US Dollars/kilogram) of producing hops varied from 5.00 for Simcoe variety to 12.10 for US Northern Brewer variety. The demand for hops has increased with the popularity of craft beer, but production has also increased. In the United States alone, planted acreage has increased from approximately 29, 000 in 2012 to 54,000 in 2016 (100). The studies reviewed here all tested hops cones, that would otherwise be used to brew beer, or food-grade extracts. The *in vivo* animal trials showed positive results on animal performance only at the highest β-acid inclusion rates tested (94, 96), which gives little hope for optimization of lower dosing rates.

Many phytochemicals, like vanillin, can be synthesized more cost effectively than they can be grown and processed, but the practicality of producing synthetic α- and β-acids is unclear. Synthetic routes to humulone (101) and lupulone (102) have been published, but industrial-scale production of these compounds does not seem readily available. The desirability of synthesizing a single compound is uncertain because the hop extracts used in many of the aforementioned studies contain a mixture of compounds, and these may act synergistically to provide a greater benefit than would be derived from a large amount of a single α- or β-acid. Another option is utilization of byproducts from breweries. Brewery byproducts have long been fed to livestock (103), but they have not been evaluated in terms of residual biologically active plant secondary metabolites. Bryant and Cohen (104) recently identified spent yeast from American craft breweries that had combined α- and β-acid concentrations in excess of 2.5 mg/g. *In vitro* experiments with rumen microorganisms revealed that the spent brewers’ yeast contained enough hops secondary metabolites to suppress ammonia production (105) and methane production (106). These results suggest that brewery waste streams could be used to provide hops phytochemicals for ruminants and other livestock.

Beyond the direct benefit to ruminant industries, we believe that hops secondary metabolites, particularly the β-acids, are useful as model phytochemical antimicrobial growth promoters. Other phytochemicals act as antimicrobial growth promoters in ruminants. For example, red clover (*Trifolium pratense*) isoflavones promote growth through antimicrobial action on the rumen HAB (107). However, isoflavones do not have an ionophore-like mechanism of action (108). When the mechanism of action and spectrum of activity against rumen bacteria are considered, lupulone and related compounds distinctly resemble feed ionophores. These hop compounds could be thought of as “phyto-ionophores” for biological points of comparison.

This review focused on the bitter acids, particularly the β-acids, but other secondary metabolites are known to be biologically active. In particular, xanthohumol has been shown to inhibit methanogens and reduce methanogenesis by mixed rumen microorganisms (109). Xanthohumol did not alter ammonia or pH, and it appeared to selectively inhibit methanogens. Xanthohumol is a prenylated flavonoid, rather than a bitter acid (73). The antimicrobial mechanism of action on rumen microorganisms has not been elucidated, but the spectrum of activity appears to be distinct from ionophores because there was no effect on ammonia concentration or pH (109). Other flavonoids are known to inhibit rumen HAB and amylolytic bacteria (84, 110). Thus, xanthohumol might have a mechanism of action that is different from either bitter acids or other flavonoids. The differences between plant secondary metabolites, even within the hops plant, and the interactions between these compounds require further investigation.

## Author Contributions

MF was primarily responsible for the manuscript, wrote sections of the manuscript, and asked the other authors to participate. IK, YW, and NN wrote sections of the manuscript.

## Disclaimer

Proprietary or brand names are necessary to report factually on available data; however, the USDA neither guarantees nor warrants the standard of the product, and the use of the name by the USDA implies neither approval of the product nor exclusion of others that may be suitable. USDA is an equal opportunity employer.

## Conflict of Interest Statement

The authors declare that the research was conducted in the absence of any commercial or financial relationships that could be construed as a potential conflict of interest.
